# Nectin like-5 overexpression correlates with the malignant phenotype in cutaneous melanoma

**DOI:** 10.18632/oncotarget.594

**Published:** 2012-08-26

**Authors:** Valentina Bevelacqua, Ylenia Bevelacqua, Saverio Candido, Evangelia Skarmoutsou, Alfredo Amoroso, Claudio Guarneri, Angela Strazzanti, Pietro Gangemi, Maria C. Mazzarino, Fabio D'Amico, James A. McCubrey, Massimo Libra, Grazia Malaponte

**Affiliations:** ^1^ Section of pathology and Oncology, Department of Bio-medical Sciences, University of Catania, Catania, Italy; ^2^ Section of Plastic Surgery, Department of Medicine and Surgery Specialities, University of Catania, Italy; ^3^ Section of Dermatology, Department of Social Territorial Medicine, University of Messina, Messina, Italy; ^4^ Department of Surgery, Azienda Ospedaliero-Universitaria Vittorio Emanuele-Ferrarotto-S. Bambino, Catania, Italy; ^5^ Pathology Unit, Azienda Ospedaliero-Universitaria Vittorio Emanuele-Ferrarotto-S. Bambino, Catania, Italy; ^6^ Brody School of Medicine at East Carolina University, Department of Microbiology & Immunology, Greenville, NC, USA

**Keywords:** NECL-5, melanoma, nevus, skin

## Abstract

NECL-5 is involved in regulating cell–cell junctions, in cooperation with cadherins, integrins and platelet-derived growth factor receptor, that are essential for intercellular communication. Its role in malignant transformation was previously described. It has been reported that transformation of melanocytes is associated with altered expression of adhesion molecules suggesting the potential involment of NECL-5 in melanoma development and prognosis. To shed light on this issue, the expression and the role of NECL-5 in melanoma tissues was investigated by bioinformatic and molecular approaches. NECL-5 was up-regulated both at the mRNA and the protein levels in WM35, M14 and A375 cell lines compared with normal melanocytes. A subsequent analysis in primary and metastatic melanoma specimens confirmed “in vitro” findings. NECL-5 overexpression was observed in 53 of 59 (89.8%) and 12 of 12 (100%), primary melanoma and melanoma metastasis, respectively; while, low expression of NECL-5 was detected in 12 of 20 (60%) benign nevi. A significant correlation of NECL-5 overexpression was observed with most of known negative melanoma prognostic factors, including lymph-node involvement (P = 0.009) and thickness (P = 0.004). Intriguingly, by analyzing the large series of melanoma samples in the Xu dataset, we identified the transcription factor YY1 among genes positively correlated with NECL-5 (r = 0.5). The concordant computational and experimental data of the present study indicate that the extent of NECL-5 expression correlates with melanoma progression.

## INTRODUCTION

Cutaneous melanoma represents the most aggressive and lethal malignancy of the skin. Despite advances in melanoma treatment [1-4], mortality from melanoma is still increasing [5]. Melanoma prevention and early detection represent the best approaches to survive [6]. Discovery of biomarkers and their application, in conjunction with traditional cancer diagnosis, staging, and prognosis, could improve early diagnosis and patient care [7-9]. Thus, there is an urgent need to develop prognostic biomarkers that can differentiate between malignant and non-malignant skin lesions and identify melanoma patients with high-risk primary lesions to facilitate greater surveillance.

Many pathways were identified to play a role in melanoma development [10-11]. It is now well known that up- or downregulation of several adhesion molecules, such as N- and E-cadherin, MCAM (melanoma cell adhesion molecule), VCAM (vascular cell adhesion molecule), integrins, can be involved in the progression of several cancer types [12-16], including melanoma [17-19]. In this context, emphasis has recently been placed on the cell adhesion molecule, nectin like molecule-5 (NECL-5), also named NECL-5/NECL-5/CD155/ Tage4 [20-23]. Experimental studies demonstrated that up-regulated NECL-5 enhances cell movement and proliferation and that cell-cell contacts do not induce a reduction of NECL-5 at the cell surface of NIH3T3 transformed cells (mouse embryonic fibroblast cell line) by oncogenic Ki-Ras; it was also suggested that up-regulation of NECL-5, mediated by activator protein-1 (AP1) pathway, might be involved in the loss of contact inhibition in transformed cells [24-28]. Previous data showed that the down-regulation of NECL-5 in cancer cells decreases migration, proliferation and metastasis [25,29]. In the mean time, its upregulation has been observed in cancer cell lines [28-30], in human tumors such as glioblastoma, ovarian carcinoma, prostate, colorectal and lung cancer [31-35]. However, the involvement of NECL-5 in human cutaneous melanoma is still lacking. Overall, these data led us to investigate if NECL-5 expression may play a role in melanoma development and progression.

In the present study, NECL-5 expression was observed at different levels in tissue samples from normal skin, benign melanocytic nevi and primary and metastatic melanoma suggesting that NECL-5 may be a potential marker of melanoma progression.

## RESULTS

### Computational analysis of NECL-5 gene in melanoma datasets

Transcript levels were higher in primary and in metastatic melanoma samples when compared to those of normal samples by analyzing several public available melanoma datasets (Supplementary Figure 1).

### NECL-5 is overexpressed in melanoma cell lines

**Figure 1:**
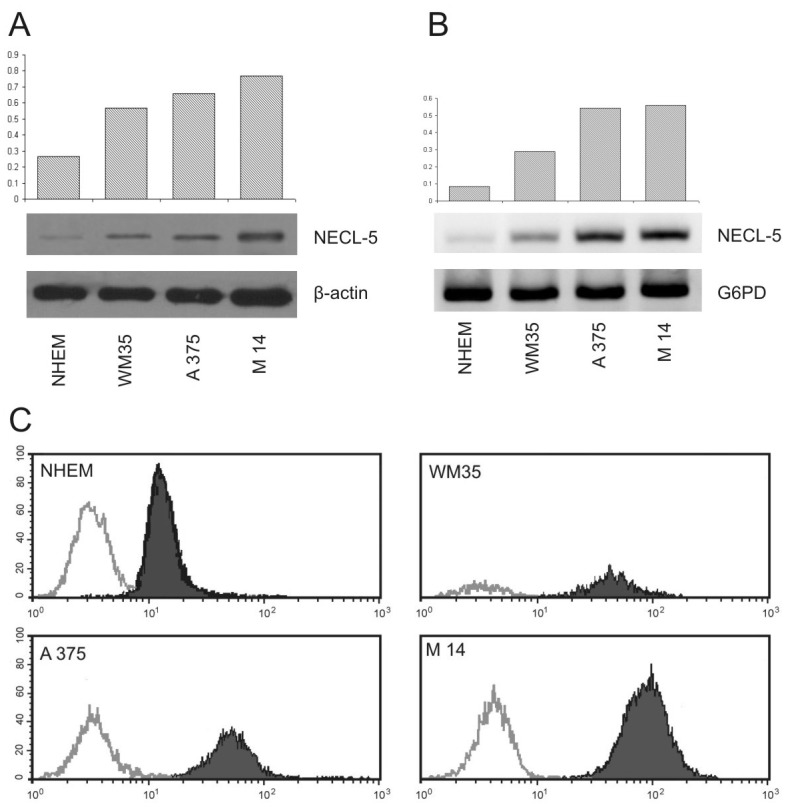
Expression analyses of NECL-5 in WM35, M14 and A375 cell lines 1A) Western blot in WM35, M14 and A375 cell lines, compared with NHEM. The upper bands represent NECL-5 protein, and the lower bands are β-Actin used as an internal control. The intensities of these bands were quantified by the Kodak 1D image analysis software. 1B) mRNA expression of NECL-5 gene by RT-PCR using G6PD for normalization. 1C) Flow cytometry analysis of NECL-5 expression. Histograms compare staining with specific antibody (black line) with the appropriate isotype control antibody (gray line).

We first analyzed the NECL-5 expression by western blot in WM35, M14 and A375 cell lines, compared with NHEM. NECL-5/β-Actin ratios were calculated for each of the three experiments and the average was used for the graph (Figure 1A). Although, western blots showed that NECL-5 is expressed in all of the cell lines, its expression in WM35 cells was significantly higher than that in NHEM cells (p< 0.001). In addition, analysis with one-way ANOVA showed that NECL-5 protein levels were significantly higher in M14 and A375 cells compared to those in WM35 cells (p= 0.002). As expected, NECL-5 mRNA levels were higher in M14 and A375 cells than in both WM35 and NHEM cells (Figure 1B). These results were also confirmed by flow cytometry analysis (Figure 1C). Finally, IHC evaluation showed that NECL-5 protein is expressed in all melanoma cell lines (Supplementary Figure 2). In detail, NECL-5 immunolabelling was mainly localized at both extracellular and intracellular level of WM35, A375 and M14 cells and a stronger immunolabelling was observed in metastatic cell lines (Supplementary Table 2).

### siRNA of NECL-5 results in decreased melamoma cell migration

**Figure 2:**
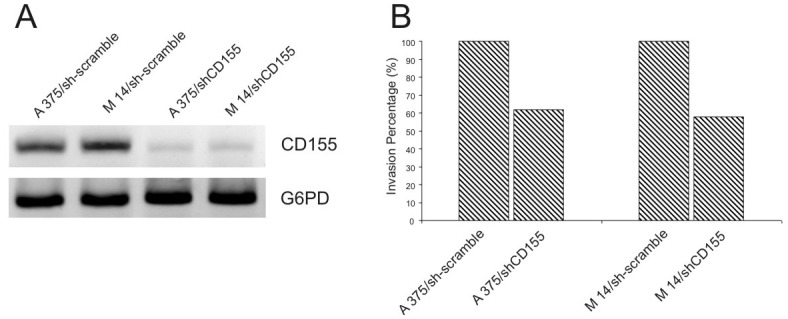
(A) siRNA of NECL-5 gene in both A375 and M14 cells was used to understand the effect on cell migration (B) siRNA of NECL-5 gene resulted in a significant decreased in transwell migration compared to control cells.

Knockdown of NECL-5 gene in both A375 and M14 cells was used to understand the effect on cell migration. As shown in Figure 2, the transfection of NECL-5-specific siRNAs cause an almost 70% loss of NECL-5 transcript levels compared to control both in A375 and M14 cells. A reduction of 40% of invasive capacity was observed when NECL-5 was knocked-down in melanoma cells compared with control cells (p < 0.001).

### NECL-5 is overexpressed in melanoma tissues

**Figure 3:**
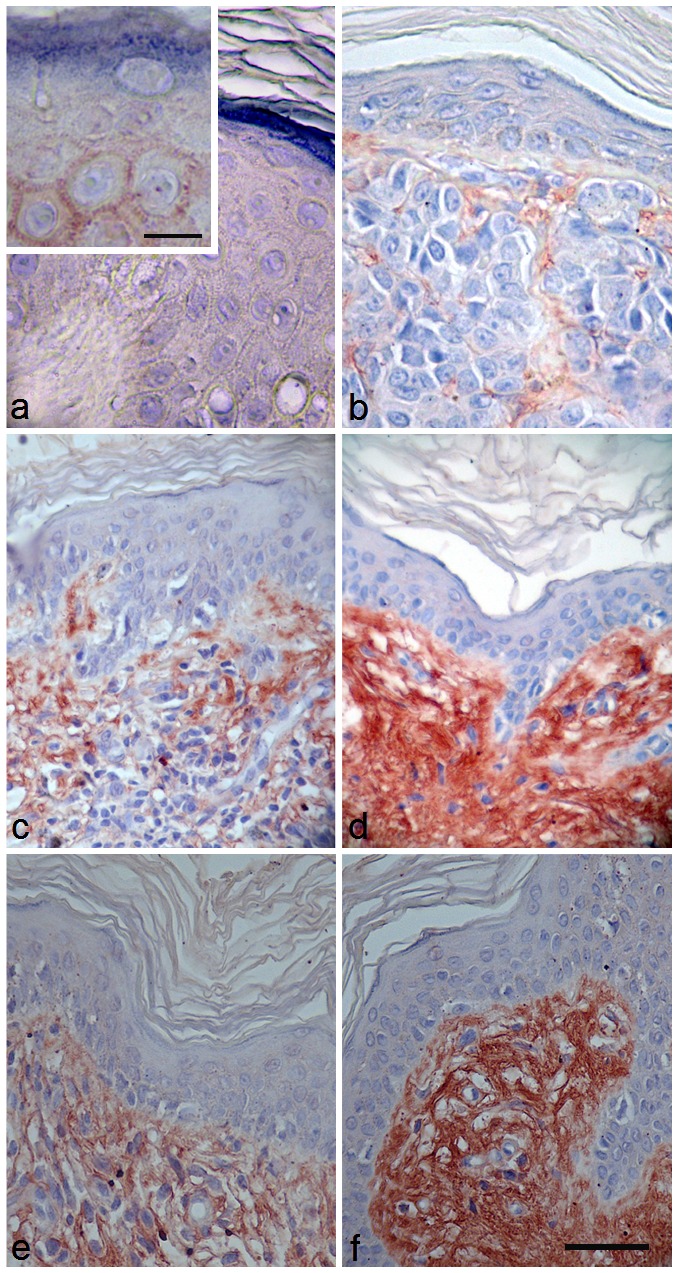
Immunostaining using a specific anti-NECL-5 antibody in a representative fraction of human melanocytic lesions of benign nevi, primary and metastatic melanoma Immunohistochemical analysis of NECL-5 in normal skin, benign nevi and melanoma tissues. a: Normal skin tissue shows no immunostaining for NECL-5 (bar: 35 μm; inset, 15 μm). b: Skin dermis of benign nevus tissue is weakly labelled for NECL-5 (bar: 32 μm). c: In primary melanoma lesion, immunolabelled melanocytes are immersed in a matrix also immunostained for NECL-5 (bar: 50 μm). d: Dermis and melanocytes of metastatic melanoma skin tissue are strongly labelled for NECL-5 molecule (bar: 50 μm). e: thickness ≤ 1 mm; f: thickness > 1 mm; NECL-5 expression is higher in melanoma sections with thickness > 1 mm in comparison with those with thickness ≤ 1 mm (bar: 50 μm).

IHC evaluation of NECL-5 was also performed in 20 benign nevi and 71 melanoma specimens, including 12 metastatic melanoma (Figure 3). Normal skin tissues showed no immunostaining (Figure 3a) whereas a positive immunostaining was in CMMs specimens (Figure 3c and 3d). The immunopositivity for NECL-5 was also significantly different between the benign (nevi) (Figure 3b) and malignant lesions (primary and metastatic melanomas) (Figure 3c and 3d). Ten of 20 (50%) benign nevi samples had minimal weak immunostaining of NECL-5. It was mainly detected in the outer zones of the rows of melanocytes and, almost absent, in cell-cell contacts between melanocytes (Figure 3b). In 3 nevi, NECL-5 immunostaining was present in the epidermis and upper dermis. Almost 91.5% of all melanoma specimens (primary and metastatic melanomas) showed an enhanced NECL-5 immunoreactivity (Figure 3c and 3d) when compared with the benign nevi tissue samples (Figure 3b) (P<0.0001). In primary melanoma, NECL-5 staining was detected in cytoplasm and/or membrane of cancer cells. Immunoreactivity of NECL-5 was observed to be higher, within tumor cells, in metastatic melanomas than in primary melanoma (P<0.001). A strongly labelled for NECL-5 there were in the papillary dermis, and variably in the reticular dermis and around blood vessels (Figure 3d). As shown in Figure 3e and 3f, NECL-5 expression is higher in melanoma sections with thickness > 1 mm in comparison with those with thickness ≤ 1 mm.

The immunoreactivity was represented by the assignment of staining index (SI), in which the intensity of immunostaining was multiplied by the extent of positive area in the tissue samples. The mean ± SE of the staining index for benign nevi was 2.3 ± 0.51; while, it was 7.95 ± 0.45 and 12.6 ± 1.58 for primary and metastatic melanoma samples, respectively (Suppl. Figure 3). In this analysis, primary and metastatic melanoma showed significantly higher level of NECL-5 expression than nevi (P<0.0001 for both). As shown in the Table 2, 8 of 20 (40%) benign nevi were negative, only 2 of 20 (10%) displayed moderate and 10 of 20 (50%) had weak NECL-5 staining. We found that 51% of primary melanomas samples showed strong (30 of 59 cases) or 27% moderate (16 of 59) or 12% weak (7 of 59) immunostaining, whereas 6 of 59 (10%) were negative and those negative samples were all melanoma *in situ*. NECL-5 was immunohistochemically detected in all melanoma metastases. Of 12 metastasis, 8 of 12 (67%) showed strong staining, 3 of 12 (25%) moderate and 1 of 12 (8%) had weak staining intensity.

**Table 1:** Socio-demographic and clinical characteristics of patients with melanoma and benign nevi

	Cases of Melanoma		Benign Nevi		χ^2^
	n	(%)	n	(%)	
Sex					
Male	42	(59)	14	(70)	
Female	29	(41)	6	(30)	P=0.38
Age (years)					
< 60	32	(45)	13	(65)	
> 60	39	(55)	7	(35)	P=0.1
Type of cancer					
Primary cutaneous melanoma including in situ melanoma	59	(83)			
Metastatic melanoma	12	(17)			
Type of primary melanoma					
Superficial spreading	53	(74.6)			
Nodular	18	(25.4)			
Tumor localization					
Head/neck	2	(2.8)	1	(5)	
trunk	50	(70.4)	13	(65)	P=0.8
extremities	19	(26.8)	6	(30)	
Breslow thickness					
≤1	39	(54.9)			
>1	32	(43.6)			
Clark's level					
I/II	38	(53.5)			
III	33	(46.5)			
Ulceration of primary melanoma					
Absent	55	(77.5)			
Presente	16	(22.5)			
Sentinel lymph node (SLN)					
Negative	59	(83.3)			
Positive	12	(16.7)			

**Table 2:** The staining intensity in melanocitic lesion

Melanocytic lesions	Immunoscore			
	Strong	Moderate	Weak	Negative
Benign nevi	-	2/20 (10%)	10/20 (50%)	8/20 (40%)
Primary melanoma	30/59 (51%)	16/59 (27%)	7/59 (12%)	6/59 (10%)
Metastatic melanoma	8/12 (67%)	3/12 (25%)	1/12 (8%)	-

### Correlation of NECL-5 expression with prognostic features in melanoma

**Figure 4:**
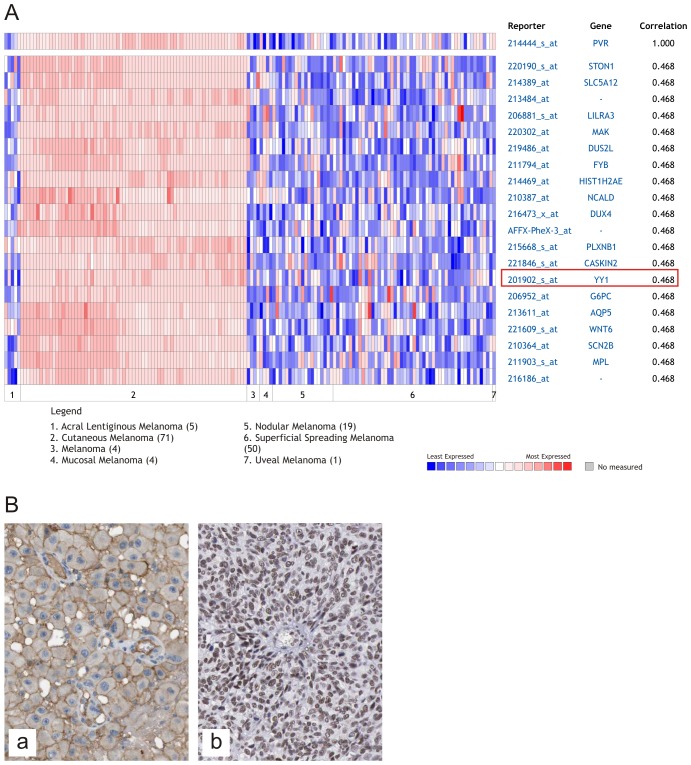
Correlation of YY1 with NECL-5 in melanoma Heat map of genes positively correlated with NECL-5 (r = 0.5) by Pearson correlation analysis in the Xu melanoma dataset (A). Immunohistochemistry evaluation of NECL-5 and YY1 in a representative case of melanoma respectively in Panel (a) and (b) (B).

We further examined a possible correlations between NECL-5 expression profiles in melanoma samples with their clinicopathologic features. Table 3 shows the potential association of NECL-5 expression levels, stratified according to the median NECL-5 immunoscore (< 9 and ≥ 9) with different clinicopathological features. As shown in Table 3, our analysis in melanoma tissue sections revealed that NECL-5 expression was strongly correlated with lympho node involvement (P = 0.009) and Breslow thickness (P = 0.004). Lymph node spreading represents the most common way of metastasis in CMMs. As expected, the comparison between the group of melanoma sections with thickness > 1 mm and those with thickness ≤ 1 mm evidenced that NECL-5 immunoreactive scores were higher in thick (score: mean 10.53 ± 0.74, P = 0.003) than in thin melanomas (score: mean 7.51 ± 0.59) (Supplemnetary Figure 4a). In particular, 81.2% (26 of 32) thick melanoma displayed 4 + of staining, 9.4% (3 of 32) had 3+ and 9.4% (3 of 32) showed 2+, while, 28.2% (11 of 39) thin melanoma had 4+, 25.6% (10 of 39) had 3+ and 46.1% (18 of 39) showed 2+ of NECL-5 staining. NECL-5 immunoscore was directly correlated with Breslow thickness (r = 0.59, P = 0.0001; Supplemnetary Figures 4b and 4c). These observations suggested a correlation between increased NECL-5 expression and clinical progression in melanoma. However, no evident correlations were observed between NECL-5 expression profiles and other clinicopathologic features, including age, sex, type of primary melanoma (superficial-spreading melanoma and nodular melanoma), Clark's levels and ulcerations.

### Positive correlation of NECL-5 with YY1 in melanoma

To further confirm the association of NECL-5 with a more malignant phenotype in melanoma development, a positive correlation between NECL-5 and YY1 transcript levels has been identified by analyzing Oncomine software (r=0.468) (Figure 4a). In agreement with computational identification, immunohistochemistry evaluation reveals that both NECL-5 and YY1 were concomitantly overexpressed in melanoma samples (Figure 4b).

## DISCUSSION

Cutaneous melanoma is the example of tumor type which progresses through various stages, from benign nevi to metastatic cancer, culminating in an highly aggressive disseminating tumor [40].

To our knowledge, this is the first report showing the expression of NECL-5 in primary and metastatic melanoma tissues and its correlation with some clinicopathologic features by using several independent approaches. These findings suggest its role in melanoma progression. A differential expression of NECL-5 was detected between primary melanoma tissues and benign nevi. Only 60% of nevi displayed minimal or weak NECL-5 expression, whereas 91.5% of melanoma specimens showed a high NECL-5 expression. The immunopositivity for NECL-5 protein was significantly different between the benign (nevi) and malignant (*in situ*, invasive and metastatic melanomas) lesions; whereas it was absent in normal skin. In benign nevi NECL-5 expression was mainly present in the outer zones of the rows of melanocytes and seemed to be almost absent in cell-cell contacts between melanocytes, even if in three nevi NECL-5 immunostaining was present in the epidermis and upper dermis and declining with increasing depth. It might speculate that NECL-5 expression in nevus cells enables them to invade into the dermis. Benign and atypical nevi have been shown to exist in clinical and histologic contiguity with cutaneous melanoma suggesting that these melanocytic nevi are also susceptible to malignant transformation [41]. These findings are consistent with other published reports describing the hypotetical role of NECL-5 overexpression in different tumors [29,31-35]. Most recently, Nakai et al. reported that NECL-5 expression is up-regulated in lung adenocarcinoma and has a negative effect on the prognosis of patients [35]. Previously, Ochiai et al. had shown that CD155 expression was up-regulated in several primary breast tumors [30]. Similarly, Sloan et al. observed that elevated espression of CD155 was detected in several primary cancer types [29]. In the current study, knockdown of the gene encoding NECL-5 in A375 and M14 cells caused a reduction of nearly 40% in tumor invasion capacity and provided further data that underline the role of NECL-5 in the progression of malignant melanoma.

Our analysis in melanoma tissue speciments showed that the increase in NECL-5 immunoreactivity correlated with lymph node involvement and Breslow thickness that are considered to be major predictive factors in melanoma microstaging [45,46]. These results revealed that increased NECL-5 expression correlated with more aggressive behaviour. In fact, high NECL-5 immunostaining in the nuclei and cytoplasm of cancer cells was detected in approximately more then 50% and 83% of primary melanomas and metastatic samples, respectively. In agreement with our findings, previous study showed that transformed cells gain metastatic ability owing to upregulation of NECL-5 [26,28]. Many studies have demonstrated that overexpression of NECL-5 is implicated in the loosening of contact inhibition and in the loss of epithelial integrity, so this enhances cell proliferation and migration may contribute to the dissemination of cells from tumors [28]. Other substrates of NECL-5 have been associated with cancer development and tumor progression. The loss of E-cadherin in melanocytic lesions would be found initially in nevi and more frequently in melanomas, this process appears to be one of the critical steps in the progression of melanoma [47]. The loss of E-cadherin in melanoma cells could trigger the release of cancer cells from the primary tumor by subsequent breakdown in the melanocytes–keratinocytes interaction and invasion of melanoma cells [47]. This suggests that increased expression of NECL-5 may lead to elevated shedding of E-cadherin and loss of cell–cell contacts. E-cadherin-mediated cell adhesion might be abrogated through degradation of the extracellular portion by gelatinases, as matrix metalloproteinases (MMPs)-2 [48]. Interestingly, it has been showed a novel link between NECL-5 and MMP-2 expression in glioblastoma [49]. This study suggested that NECL-5 enhance PI3K/Akt signalling at the leading edge during migration. The signaling guide to MMP-2 production, which can facilitate invasion. MMP-2 is strongly expressed in malignant melanomas and it correlates with invasion and metastatic behavior [50]. Thus, up-regulation of NECL-5 expression may be a common pathway through which cancer cells can acquire a more invasive or dispersive phenotype, a requirement for cancer progression [51].

Furthermore, the implication of NECL-5 with the aggressiveness of melanoma may be corroborate by its correlation with the transcription factor YY1. Our recent data showed that transcript levels of the transcription factor YY1 are higher in several cancer types including melanoma when compared with those of normal tissue [52-55]. We have also observed that the inhibition of YY1 in A375 melanoma cells induces p53-mediated apoptosis after treatment with nitric-oxide donor [56]. These observations have lead us to investigate if YY1 transcript levels correlate with NECL-5 mRNA levels in melanoma. As aspected, by analyzing the large series of melanoma samples in the Xu dataset, we observed that YY1 is positively correlated with NECL-5 (r = 0.5) underlying that its overexpression is associated with a malignant phenotype in melanoma development.

In conclusion, we have provided evidence that NECL-5 may be an important biomarker in the early diagnosis of melanoma, being slightly expressed already in benign nevi. Moreover, NECL-5 seems to be an indicator of tumour progression in melanoma. NECL-5 is strongly expressed in metastatic melanoma cell lines. In primary melanoma lesions was its expression was localized in the deeper part of the tumors and was consistent with a possible role in the progression toward an invasive phenotype. Limitations of our study is the absence of clinical follow-up, including survival data. Additional studies of the spectrum of expression of NECL-5 and its relationship with other adhesion molecules and growth factors will be necessary to address further the complex processes of pathogenesis and progression in malignant melanoma. Our future goals will be to determine the molecular and cellular consequences as the result of an increased NECL-5 expression. These studies will allow the linking of NECL-5 to specific signalling pathways so that rational mechanism-based therapeutic strategies directed at specific molecular targets can be developed toward the potentially better diagnosis and management of melanoma.

## MATERIALS AND METHODS

### Gene expression data sets

Computational evaluation of NECL-5 has been performed by ONCOMINE software. Differential mRNA expression analyses among normal skin tissue versus benign melanocytic skin nevus (BMN), BMN versus cutaneous malignant melanoma (CMM) and CMM versus metastatic melanoma were explored in 3 datasets showing statistical significance less than 0.05 (p<0.05) [36-38]. Statistical analysis was accomplished through use of ONCOMINE algorithms. Haqq Melanoma Dataset was excluded from the study as a different microarray platform has been used by the authors [39]. Fold change values were considered for this analysis. Further details on datasets are reported in Supplementary Table 1. Correlation between NECL-5 and Yin Yang 1 (YY1) transcript levels was analyzed according to Oncomine software in the Xu Melanoma Dataset [38].

### Cell lines and culture conditions

Melanoma cell lines (WM35, A375, M14) and human epidermal melanocyte (NHEM) cell line were kindly provided by Dr.V. Russo (Tumor Targeting Research Unit, San Raffaele Scientific Institute, Milano, Italy) and Dr. R. De Maria (Department of Hematology, Oncology and Molecular Medicine, Istituto Superiore di Sanità, Rome, Italy), respectively. Melanocytes and melanoma cell lines were maintained in Melanocyte Growth Medium (Lonza, Walkersville, USA) and RPMI medium (Gibco, Life Technologies Inc., Milan, Italy), supplemented with 2 mmol/l L-glutamine, 100 IU penicillin and 100 μg/ml streptomycin and 2% heat-inactivated fetal calf serum (Gibco Life Technologies Inc.), respectively. All cell lines were cultured at 37°C in 5% CO_2_ atmosphere at constant humidity and passaged twice a week. Melanocytes and melanoma cells were grown to subconfluency, harvested and subjected to westen blot, real-time polymerase chain reaction (RT-PCR) or flow cytometry and immunoistochemistry analysis (IHC).

### Tissue samples and patients characteristics

Archival paraffin tissue section of 91 melanocytic lesions excised between 2006 and 2010 were retrieved from the Department of Pathology at the Vittorio Emanuele Hospital, Catania, Italy. The collection and storage of samples were performed according to local ethical guidelines. Institutional review board approval was obtained for this study. There were (i) 59 CMM, including 10 melanoma *in situ*, (ii) 12 metastatic malignant melanoma, randomly chosen for the study, (8 subcutaneous melanoma lesions and 4 regional lymph node metastases), (iii) 20 BMN, (iv) 10 normal skin adjacent to different tumour masses. Clinical staging of CMM was performed on a pathological basis according to the new American Joint Committee on Cancer 2001 classification system. Diagnosis and staging of BMN had been performed on the basis of clinical, histopathologic findings. All sections of CMM were superficial spreading melanoma (SSM) and nodular melanoma (NM), at different stages (Clark's level I to III). Localization of the primary melanoma were grouped in three classes: head/neck, trunk, extremities. We stratified CMM by the Breslow tumor thickness as thin (≤1mm) and thick (>1mm). Specimens were fixed in neutral 4% buffered formaldehyde for a minimum of 24h and subsequently embedded in paraffin. The clinicopathological features of 91 melanocytic lesions are summarized in Table 1.

### RT-PCR analysis

Total cellular RNA was extracted from cultured cell lines with Micro-to-Midi total RNA purification system (Invitrogen, Milano, Italy) according to the manufacture's instructions. Reverse transcription was carried out using M-MLV reverse transcriptase (Invitrogen) and random primers (Invitrogen). Semiquantitive PCR was carried out by applying standard condition. The following primers were used (5’→3’): CD155 sense: TATCTGGCTCCGAGTGCTTGCC; CD155 antisense ACGACGGCTGCAAAAGTGGCG; glucose-6-phosphate dehydrogenase (G6PD) sense: ACGTGATGCAGAACCACCTACTG; G6PD antisense: ACGACGGCTGCAAAAGTGGCG. For quantitation, gels were scanned, and the pixel intensity for each band was determined using the Image J program and normalized to the amount of G6PD.

### Immunoblot Analysis

Immunoblot analysis was performed as described by Merrill et al.[31]. Antibodies included anti-CD155 (ab 103630, Abcam, Cambridge, UK) and anti-β Actin (Sigma-Aldrich). CD155 antibody binding was detected with streptavidin-horseradish peroxidase complex (Roche, Indianapolis, IN) and visualized using enhanced chemiluminescence substrate (Amersham Life Science, Piscataway, NJ, USA). β Actin was used as an internal loading control.

### CD155 siRNA

A double stranded siRNA oligonucleotide targeting CD155 (5’-CAACUUUAAUCUGCAACGUdTdT-3’) was chemically synthesized (Dharmacon Research) and transfected into WM35, A375 and M14 cells using Oligofectamine (Invitrogen) following manufacturers instructions using 200 nM siRNA per 10 cm dish. Cells were incubated with siRNA in OptiMEM (Invitrogen) for 6 hrs after which time normal growth media was added. Cells were then incubated for 72 h to achieve >80% knockdown of CD155. Control cells were transfected with a scrambled siRNA oligonucleotide at matching concentration. After transfection, cells were harvested and subjected to the invasion assay.

### Tumor invasion assay

The in vitro invasion assay was done by using a cell invasion assay kit based on the manufacturer's protocol (Chemicon, Billerica, MA). Control cells and siRNA-treated cells were trypsinized and used for the invasion assay.

### Immunohistochemistry

Immunohistochemical staining of NECL-5 were done according to standard protocols using CD155 antibody (ab60115, Abcam, Cambridge, UK). Detection of primary antibody was performed using the streptavidin–biotin–peroxidise complex system (Santa Cruz Biotechnology Inc., Santa Cruz, CA)., according to the manufacturer's instructions. The detection was carried out using Histostain-Plus Kit (Zymed, South San Francisco, CA). AEC (3-amino-9-ethylcarbozole) was used as a chromogen. Negative control experiments were performed as above described. Detection of YY1 was performed by using Sigma Aldrich HPA001119 antibody according to the manufacturer's instructions.

### Image analysis

Immunolabelled and sampled tumor sections were observed using a Leica DMRB microscope (10× and 40× magnification) (Leica, Wetzlar, Germany), the images were photographed with a Canon G-9 camera (Canon, Japan) and analyzed using Image J software. Four randomly chosen fields of view were assessed in the melanoma biopsies and in the cell lines. A section was considered negative or positive according to the absence or presence of cytoplasmic staining. Immunoreactivity was assessed by the amount of positive malignant cells in the area. We used a staining index (SI; values 0-16) with the following formula: SI = immunostaining intensity × positive area, where intensities were scored semiquantitatively as follows: 4+, very strong; 3+, strong; 2+, moderate; 1+, weak and 0, negative. Positive areas were defined as the epithelial area that showed positive malignant cells for NECL-5 immunoreactivity and was graded on an arbitrary scale as 0, absent; 1+, less than 25%; 2+, 25% to 50%; 3+, 51% to 75%; and 4+, more than 75%. The distribution of staining was classified as focal or diffuse. Localization of staining (membranous or cytoplasmic) was also recorded. Scoring was based upon the consensus of three anatomic pathologists.

### Flow cytometry analysis

Cell lines were washed in PBS. Cell suspension were stained with phycoerythrin conjugated mouse IgG1 isotype control and mouse IgG1 anti-CD155 (Bioscience, USA). Cells were analyzed using a FACScalibur flow cytometer (Becton Dickinson, USA). After gating cells on a forward scatter/side scatter dot plot window on linear scale, the fluorescence intensity of PE-conjugated isotype control and anti-CD155 labelled cells were analyzed in histograms on FL2 channel with logarithmic scales.

### Statistical analysis

The statistical analyses were performed using SPSS for Windows. Comparison of socio-demographic and clinical characteristics benign nevi and melanoma patients was performed with the χ^2^ test. Normally distributed data were expressed as media ± SE and non normally distributed data were expressed as median (range). To correlate NECL-5 staining index and clinicopathologic features was applied the χ^2^ square test. Additionally, the correlations between NECL-5 expression in melanoma sections and Breslow thickness were assessed by Spearman rank correlation. For semiquantitative RT-PCR analysis, statistics was performed by using the unpaired Student's *t* test. The statistical differences of NECL-5 expression level between primary and metastatic melanoma samples were calculated by Student *t* test or one-way analysis of variance (ANOVA). Differences were considered to be statistically significant at a level of p < 0.05.

## Supplementary Figures and Tables




