# Supplementary material for: Nectin like-5 overexpression correlates with the malignant phenotype in cutaneous melanoma
Source: Oncotarget. 2012 Aug 26;3(8):882–92. doi: 10.18632/oncotarget.594 (PMC3478464; doi:10.18632/oncotarget.594)
Supplement: Supplementary file 1 [file oncotarget-08-882-s001.pdf]

Nectin like -5 overexpression correlates with the malignant phenotype in cutaneous melanoma - Valentina Bevelacqua et al

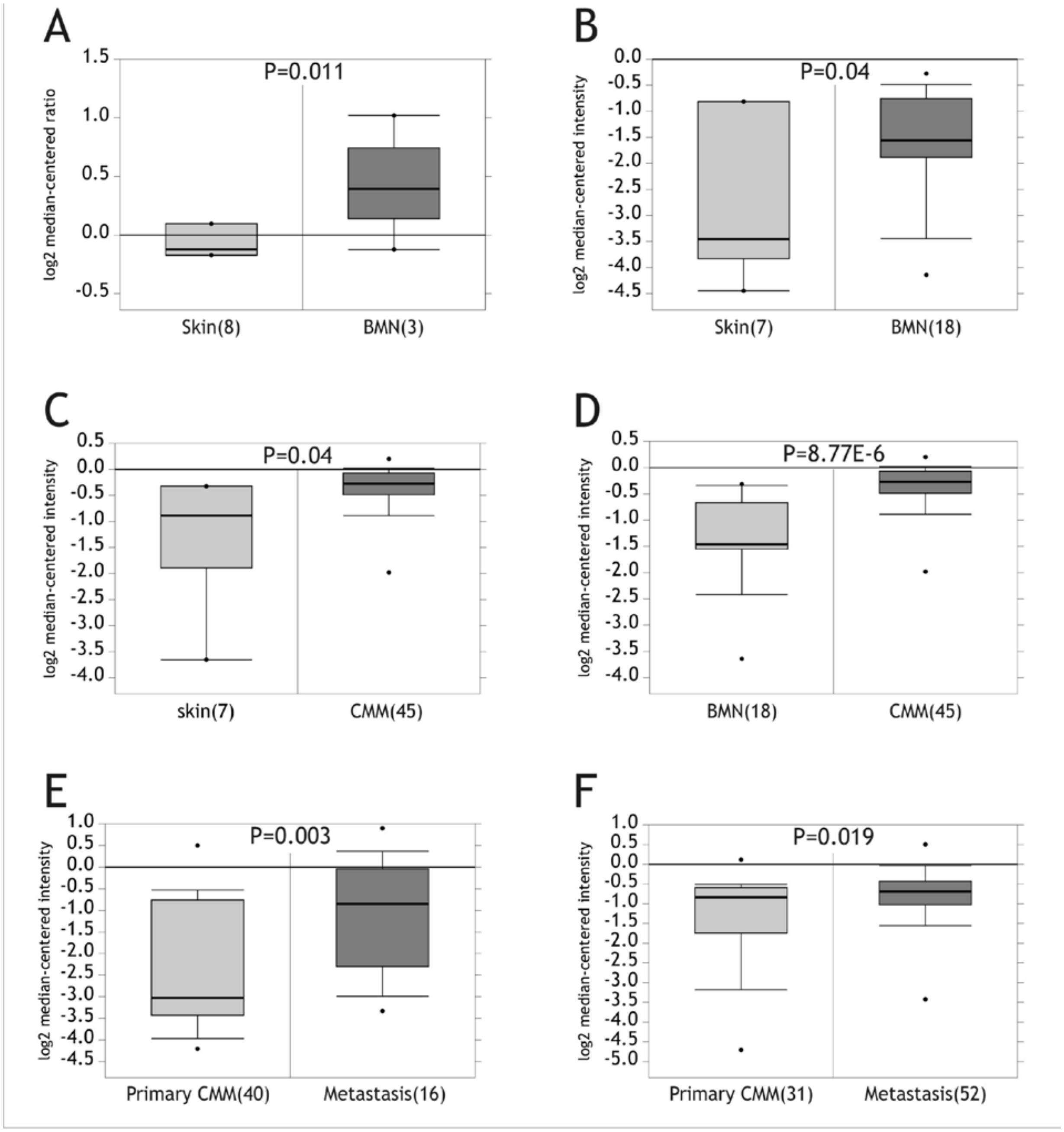

Supplementary Figure 1: Bioinformatic analysis of NECL-5 by ONCOMINE software in different publicly available datasets (see Supplementary Table 1). Differential mRNA expression analyses among normal skin tissue versus benign melanocytic skin nevus (BMN) (A and B); normal skin versus melanoma cutaneous malignant melanoma (CMM) (C); BMN versus CMM (D); Primary melanoma versus metastic melanoma (E).

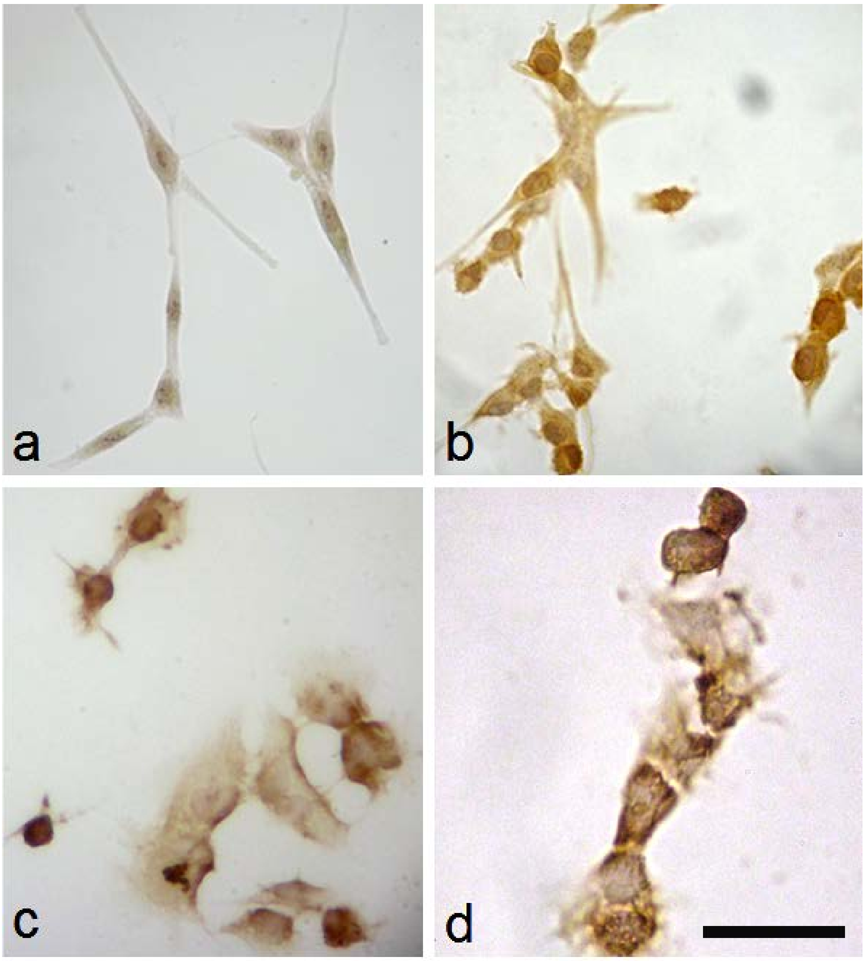

Supplementary Figure 2: Immunolabelling for NECL-5 protein in normal melanocytes and melanoma cell lines. In NHEM (a), the immunostaining is almost absent. MW35 (b), A375 (c) and M14 (d) cells are discretely labelled for NECL-5. The protein is mainly localized on cell membrane as well as on the cell leading edge (bar: 50 µm).

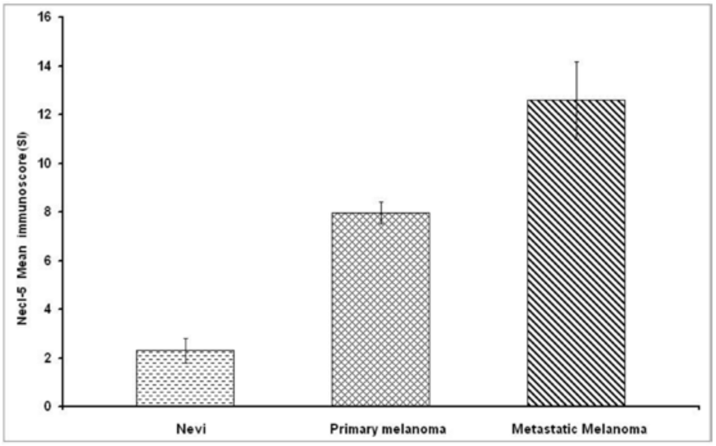

Supplementary Figure 3: The mean ± SE of the staining in benign nevi, primary and metastatic melanoma samples. Both primary and metastatic melanoma showed significantly higher level of NECL-5 expression than nevi (P<0.0001).

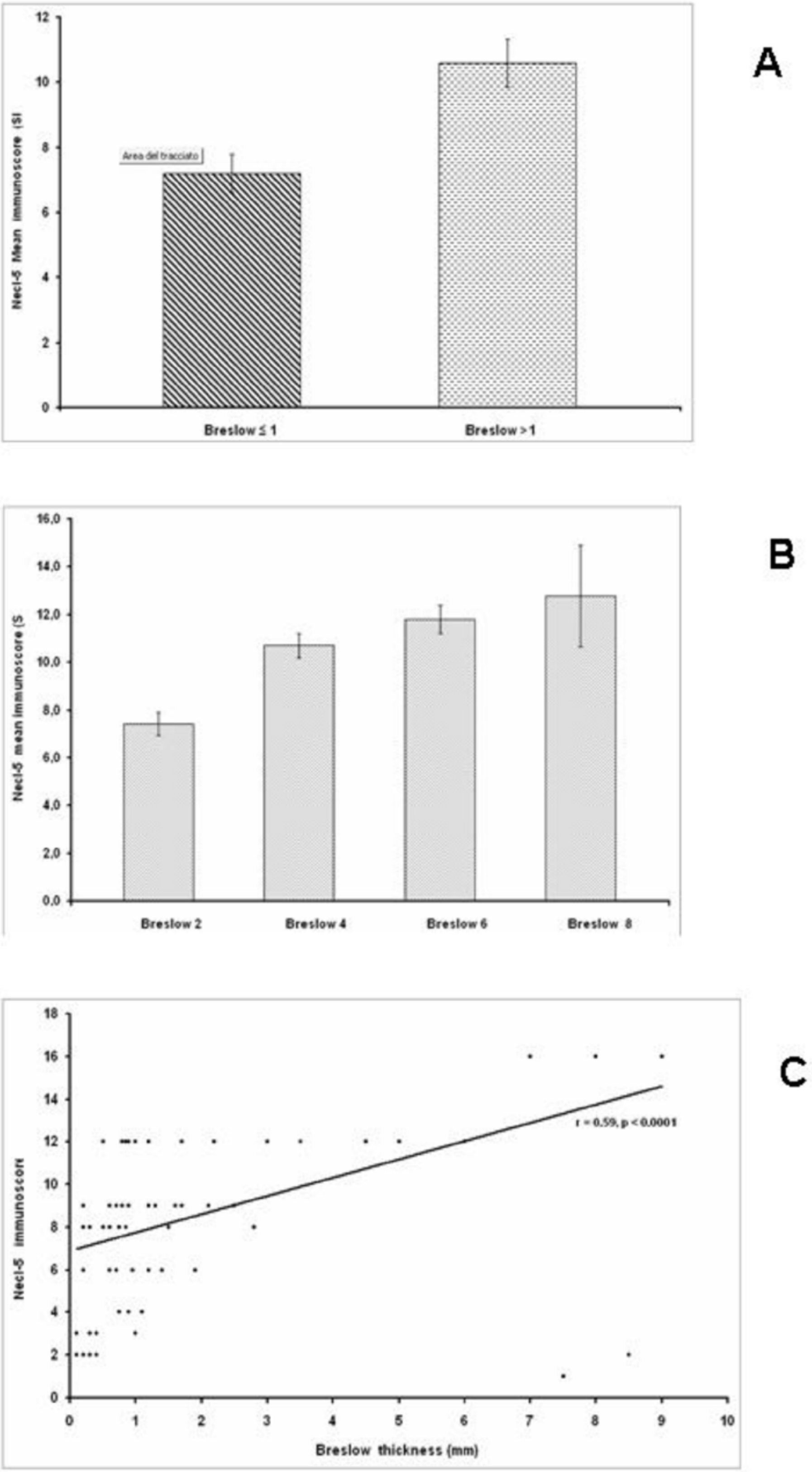

Supplementary Figure 4: Differences of NECL-5 immunoreactive scores in melanoma samples with Breslow ≤ 1 mm and those with thickness > 1mm. NECL-5 immunoreactive scores were higher in thick (score: mean 10.53 ± 0.74, P = 0.003) than in thin melanomas (score: mean 7.51 ± 0.59) (4a). NECL-5 immunoscore in different Breslow levels of melanoma samples (4b). Correlation between NECL-5 expression and Breslow tumor thickness by Spearman rank test (r = 0.59, P = 0.0001) (4c).
