# Supplementary material for: Nectin like-5 overexpression correlates with the malignant phenotype in cutaneous melanoma
Source: Oncotarget. 2012 Aug 26;3(8):882–92. doi: 10.18632/oncotarget.594 (PMC3478464; doi:10.18632/oncotarget.594)
Supplement: Supplementary file 2 [file oncotarget-08-882-s002.pdf]

Nectin like -5 overexpression correlates with the malignant phenotype in cutaneous melanoma - Valentina Bevelacqua et al

Supplementary Table 1: Fold change values of PVR gene expression

**Analysis Type**	**N. of samples**		**fold change**	**P < 0.05 (T-test)**	**Data set**		
					**Author (ref.)**	**Year**	**Platform**
Skin vs BMN	normal(7)	nevus(18)	2.227	0.04	Talantov D *et al*	2005	U133A
Skin vs CMM	normal(7)	cancer(45)	1.896	0.04	Talantov D *et al*	2005	U133A
BMN vs CMM	nevus(18)	cancer(45)	2.055	8.77E-06	Talantov D *et al*	2005	U133A
Primary CMM vs Metastasis	primary(16)	metastasis (40)	2.384	0.003	Riker AI *et al*	2008	U133A plus 2.0
Primary CMM vs Metastasis	primary(31)	metastasis (52)	1.394	0.019	Xu L *et al*	2008	U133A

Supplementary Table 2: Comparison expression of NECL-5 in melanoma cell lines

Melanoma cell lines	Intensity and percentage of positive cells in melanoma cell lines
Normal melanocytes	+/+
WM35 (primary melanoma)	+++/+++
A 375 (metastatic melanoma)	++++/++++
M14 (metastatic melanoma)	++++/++++

Intensity of immunostaining was graded as follows: 4+, very strong; and 1+, weak, and 0, no staining. Intensity score was graded as follows: 4+, diffuse staining of all melanocytes, 3+, about 75% and 2+, about 50 % and 1+, less than 25%, 0, negative.
